# The effect of cortex eucommiae on alleviating lung injury induced by *Haemophilus paragallinarum*

**DOI:** 10.1016/j.psj.2025.105906

**Published:** 2025-09-26

**Authors:** Bo Zhang, Bo Liao, Rou Sang, Rong Liao, Dengyao Xu, Yanhong Lv, Fangjie Li, Ke Li, Aiguo Xin

**Affiliations:** a College of Agriculture and Life Sciences, Kunming University, 650200, China; b Institute of Poultry Science, Yunnan Academy of Animal Husbandry and Veterinary Sciences, 650224, China

**Keywords:** Cortex eucommiae, *Haemophilus paragallinarum*, Pathological injury, Autophagy

## Abstract

To analyze whether Cortex Eucommiae (CE) can inhibit pathological lung injury by regulating autophagy levels in chicks lung tissue and identify potential targets, thereby alleviating pneumonia damage caused by Haemophilus paragallinarum (*Hp*) infection. Experimental groups were established: Control group, *Hp* infection group, *Hp*+CE group, *Hp*+Rapamycin (*Hp*+Rapa) group, and *HP*+(NLR family, pyrin domain-containing 3, NLRP3) inhibitor (INF39) group. Assessments included: HE staining and pathological scoring of chick lungs, ELISA measurement of inflammatory cytokine expression, RT-PCR analysis of key autophagy genes and NLRP3/Caspase-1/IL-1β inflammatory pathway genes, Immunohistochemistry and Western blot analysis of autophagy protein (microtubule-associated protein 1 chain 3, LC3) and inflammatory proteins NLRP3, Caspase-1, and IL-1β. CE reduced pro-inflammatory cytokine secretion, inhibited NLRP3, Caspase-1, and IL-1β protein expression, and upregulated key autophagy genes and proteins, thereby promoting autophagy and subsequently alleviating *Hp*-induced pneumonia damage in chicks. CE alleviates *Hp*-induced acute lung injury in chickens by promoting autophagy and suppressing NLRP3 inflammasome expression. CE may represent a potential therapeutic agent for treating bacterial-induced acute lung injury.

## Introduction

*Hp* is a significant pathogen causing respiratory diseases in chicks, prevalent in poultry farming environments (Blackall et al., 1999). It can induce various conditions, including infectious coryza, tracheitis, and pneumonia, posing a serious threat to chicks health and productivity (Clothier et al., 2019). *Hp* infection typically spreads via the respiratory route. After invading the host, the pathogen first colonizes the upper respiratory mucosa, then breaches the mucosal barrier to reach lung tissue, triggering inflammatory responses and tissue damage (Kuchipudi et al., 2021). Initially, infected birds may exhibit mild respiratory symptoms like nasal discharge and sneezing. As the disease progresses, inflammation intensifies, leading to distinct lung pathologies such as alveolar structural destruction and inflammatory cell infiltration (Poernomo et al., 2000). These pathological changes impair normal lung function and can potentially cause systemic inflammatory response syndrome (SIRS), further hindering growth and productivity (Heuvelink et al., 2018). In recent years, the incidence of *Hp* infection has risen with the expansion of intensive farming, causing substantial economic losses to the poultry industry. While traditional antibiotic treatments can partially control infections, antibiotic misuse has led to resistant strains and environmental pollution (Nouri et al., 2021). Consequently, discovering novel, safe, and effective anti-infective agents and immunomodulators is a critical research focus in poultry disease prevention.

Cortex Eucommiae is a natural plant extract rich in bioactive compounds such as chlorogenic acid, flavonoids (e.g., quercetin, kaempferol), and polysaccharides (Wang et al., 2019). Recently, CE has gained attention due to its diverse pharmacological properties, including antioxidant, anti-inflammatory, antibacterial, and immunomodulatory effects (Ye et al., 2023). Studies indicate that chlorogenic acid in CE possesses potent antioxidant capacity, scavenging free radicals and inhibiting lipid peroxidation. Flavonoids suppress inflammatory cytokine expression to mitigate inflammation, while polysaccharides enhance immune function and disease resistance (Gao et al., 2023). The potential of CE in mitigating lung injury is increasingly recognized. Research has shown that CE can alleviate lipopolysaccharide (LPS)-induced lung injury in mice through its antioxidant and anti-inflammatory actions, improving lung tissue pathology (Qiu et al., 2018). Furthermore, CE modulates the immune system, boosting host resistance to pathogens. These findings suggest promising applications for CE in preventing and treating pulmonary diseases (Cheng et al., 2022).

Complex interactions exist between cellular autophagy and the NLRP3 inflammasome, Caspase-1, and IL-1β (Luo et al., 2025). Under normal conditions, cell autophagy negatively regulates the NLRP3 inflammasome by degrading its components. This subsequently reduces the activation of Caspase-1 and the release of IL-1β and IL-18, thereby exerting an anti-inflammatory effect (Zhou et al., 2025; Boršić et al., 2025). When autophagy is impaired, increased mitochondrial ROS activates the NF-κB signaling pathway, promoting the transcription of NLRP3 and pro-IL-1β. This activates the NLRP3 inflammasome, leading to Caspase-1 activation and the maturation and release of pro-inflammatory cytokines like IL-1β (Luo et al., 2025). In pathological conditions, such as metabolic or infectious diseases, this balance is disrupted. Aberrant NLRP3 inflammasome activation causes excessive inflammation, and dysfunctional autophagy can exacerbate this process, accelerating disease progression (Danielski et al., 2018). Therefore, investigating the relationship between autophagy and the NLRP3/Caspase-1/IL-1β pathway is crucial for understanding disease mechanisms and developing novel therapeutic strategies.

In summary, while CE demonstrates anti-inflammatory, antioxidant, and immunomodulatory functions, its protective effects against bacterial lung injury remain inadequately studied. This research aims to elucidate the potential mechanisms of CE in ameliorating lung injury at the genetic and protein levels, providing a theoretical foundation for further investigation and application.

## Materials and methods

### Experimental animal

Fifty specific pathogen free (SPF) chicks (15-day-old, male, 120±10g) were purchased from Yunnan Bestai Biotechnology Co., Ltd. (Yunnan, China). All procedures were approved by the Experimental Animal Ethics Committee of Kunming University. Experiments were conducted in accordance with the Guidelines for the Ethical Review of Animal Welfare (EAE-GZU-2022-E021) and adhered to protocols approved by the Institutional Animal Care and Use Committee (IACUC).

### Haemophilus paragallinarum culture

The *Hp* strain *Hp*-8 was obtained from the China Institute of Veterinary Drug Control. *Hp* was cultured on 5% serum agar plates at 37°C for 18 hours. Single colonies were then inoculated into liquid medium for expansion. Chicks were infected via intranasal instillation of 0.3mL *Hp* suspension (OD₆₀₀_nm_=0.6∼0.8) per chick.

### Animal groups

Fifty SPF chicks were randomly assigned to five groups (n=10 per group): Control group, *Hp* group, *Hp*+CE group, *Hp*+Rapa group, *Hp*+INF39 group (a positive control for the specific inhibition of the NLRP3 inflammasome pathway). Groups 2-5 received intranasal instillation of 0.3mL *Hp*. The Control group received an equal volume of PBS intranasally. After 24 hours, treatments began via subcutaneous injection: Rapa (10mg/kg), INF39 (100mg/kg), or CE (100mg/kg) according to group assignment. Treatments continued for 7 consecutive days. Blood was then collected from the wing vein for serum separation. Chicks were euthanized via exsanguination, and lung tissues were collected for subsequent analysis.

### Histopathology

Sections of the left lung lobe were fixed in 4% paraformaldehyde, embedded in paraffin, and sectioned at 5-6 µm thickness. Sections were stained with hematoxylin and eosin (H&E), mounted with neutral gum, and examined under a light microscope. Lung injury was scored on a 0-1 scale based on the following criteria: 0 (no injury), 1 (minimal, <25% involvement), 2 (mild, 25-50%), 3 (moderate, 50-75%), and 4 (severe, >75% involvement). Parameters included alveolar congestion, hemorrhage, neutrophil infiltration, and alveolar wall thickening (Luan et al., 2021).

### ELISA

Serum levels of pro-inflammatory cytokines (TNF-α, IL-18, IL-1β) were measured using chicken-specific ELISA kits according to the manufacturer's instructions.

### RT-PCR

Total RNA was extracted from chicken lung tissue and reverse-transcribed into cDNA using a commercial kit. RT-PCR was performed to analyze the expression of key genes involved in autophagy and the NLRP3/Caspase-1/IL-1β inflammatory pathway. Relative mRNA transcription levels of target genes were calculated using the 2^-ΔΔCt^ method. Primer sequences for (molecular target of rapamycin, mTOR), (autophagy related gene, ATG)3, ATG5, ATG7, Beclin-1, LC3II, NLRP3, ASC, pro-Caspase-1, Caspase-1, pro-IL-1β, and IL-1β are listed in Table 1.

**Table 1:** Specific primers for the target gene.

Gene	Primer (5′−3′)	Annealing temperature (°C)	Fragment length
mTOR	F:AGTCGTACTGACGTCGTACGTA R:CGTACGTGCACGTGCTTGACG	59.5	158
*ATG3*	F:AGCTGCTGACGTCCGATGCG R:GCTACGACTGCTGCACGTCG	60	172
*ATG5*	F:AACGTCGACGTCACTGCGTC R:AGCTACGTACGAAGCTCGGC	58.8	98
*ATG7*	F:CGACCGTACGCTCCGACCGT R:CGCTCGTCCCACGTACCAC	59.6	104
*Beclin-1*	F:CCGATCGCTCGATGCGGTTTCC R:CCGACCGTACGTACCGTACCGT	61.4	156
*LC3II*	F:CGATCAGCTACCGTACGCT R:GGCGTACGTGGTCAACCGT	60	148
*NLRP3*	F:GTGCGTACGTCACTGTGCCA R:GCTCGTACGTACGGTCAACG	59.6	136
*ASC*	F:CGCTCGACGTCCGATCGCTAC R:CGCTGCTCGCTCGTTCGAACCG	61.5	170
*pro-Caspase-1*	F:CGCTCACACCAGTCCAGTCGAT R:GTCGCTAACACACGTCCAGTC	61	139
*Caspase-1*	F:CGTCGACACAGTCGACAGTCC R:CGTAACACACGCGCGAACAGT	60.5	158
*pro-IL-1β*	F:CCGCTCAAACCGTACCGCTAA R:CGTCGACAGTCGAGTCGATCG	59	152
*IL-1β*	F:CCGACGATCCGTACCAGATCA R:CGACCAGACAGCTACAGTCGT	58.5	164
*β-actin*	F:CGCTAACACACCGTAGCACGT R:CGACACATACAGATACAGCCC	60	102

### Immunohistochemistry

Right chick lung tissues were fixed in 4% paraformaldehyde, sectioned, and incubated with primary antibodies against LC3II (1:100), NLRP3 (1:600), Caspase-1 (1:1000), and IL-1β (1:300) (Wanlei Biotechnology Co., Ltd., Shenyang, China) at 4°C for 12h. After adding secondary antibodies, DAB staining was performed per the instructions (Shanghai YuanYe Biotechnology Co., Ltd., Shanghai, China). Sections were stained with hematoxylin and eosin, sealed with neutral gum, and observed under a microscope. Images were analyzed using Image ProPlus 6.0 software.

### Western blot

Right chick lung tissues from each group were immersed in protein lysis solution, centrifuged, and the supernatant collected. Protein concentrations were measured using a BCA kit. LC3I and LC3II expression levels in different groups were determined by Western blot, and grayscale values were analyzed using Image J software.

### Statistical analysis

Data were analyzed using SPSS21.0. Measurement data were expressed as mean±standard deviation (xˉ±s). One-way ANOVA and independent-sample t-tests were used for comparisons, with P<0.05 indicating significance and P<0.01 indicating high significance.

## Results

### CE mitigates inflammatory damage caused by Hp infection

**Fig. 1:**
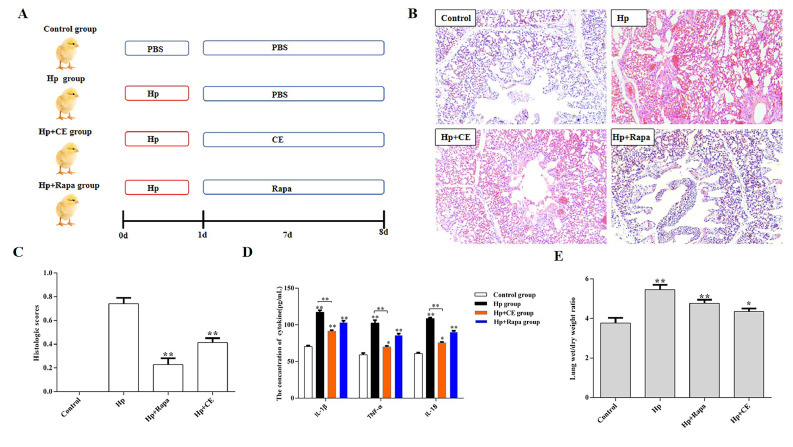
CE alleviates the inflammatory injury of the lungs in chicks caused by Hp A. Experimental Plan; B. HE staining showed the pathological changes of lung tissue in chicks (200x). C. Histopathological score (extent of the lesion) of lung tissue in chicks. D. Expression of inflammatory factors in the lungs of chicks; E. Wet/Dry weight ratio of lung tissue in chicks. (*P<0.05, **P<0.01).

Lung pathological changes, as assessed by HE staining, revealed no visible alterations in the control group. In contrast, the *Hp* group exhibited marked pulmonary pathology, characterized by massive inflammatory-cell infiltration, thickening of alveolar walls, narrowing of alveolar spaces, and capillaries engorged with red blood cells (Fig. 1**B**). Notably, intervention with CE and Rapa significantly improved the histopathological changes in the lungs of chicks in both the CE+*Hp* and *Hp*+Rapa groups (Fig. 1**C**).

ELISA analysis of serum samples demonstrated that the *Hp* group had significantly elevated levels of the pro-inflammatory cytokines IL-1β, IL-18, and TNF-α compared to the control group (P<0.01). However, following treatment with CE and Rapa, the levels of these cytokines were markedly reduced in both the *Hp*+CE and *Hp*+Rapa groups (P<0.01) (Fig. 1**D**). These findings suggest that CE has the potential to reduce the secretion of pro-inflammatory factors, indicating its suppressive effect on *Hp*-induced pulmonary inflammation in chicks.

The wet/dry weight ratio of lung tissues, indicated that *Hp* infection led to a significant increase in the lung wet/dry weight ratio in chicks compared to the control group (P<0.01). Conversely, chicks treated with CE and Rapa exhibited a decrease in this ratio (Fig. 1**E**). These results imply that *Hp* induces serous exudation in lung tissue cells, whereas CE can effectively inhibit this *Hp*-induced exudation.

### CE impact on autophagy and oxidative stress

**Fig. 2:**
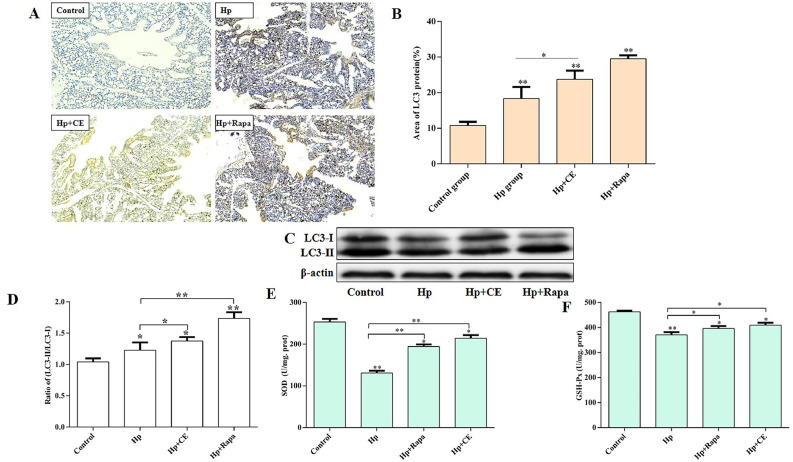
CE Impact on Autophagy and Oxidative Stress Indicators (A) Assessment of the effect of Hp infection on the autophagy marker protein LC3 in chicken lungs using immunohistochemistry (200x). (B) Rate of autophagy-positive cells. (C) Detection of LC3I and LC3II protein expression in chicken lungs by Western blot. (D) Ratio of LC3I to LC3II. (E) SOD antioxidant activity. (F) GSH-Px antioxidant activity. (*P<0.05, **P<0.01).

We used immunohistochemistry and Western blotting to detect the effect of CE on autophagy, and assessed the effects of CE on the oxidative stress markers GSH-Px and SOD using kits. The results showed that compared with the control group, the autophagy rate of lung tissue cells after *Hp* infection was significantly increased (P<0.01). Compared with the *Hp* group, the autophagy rate of the *Hp*+CE and *Hp*+Rapa groups was significantly increased (P<0.01) (Fig. 2**A, B, C, D**). Compared with the control group, the antioxidant activity of GSH-Px and SOD in the *Hp* group was reduced, while compared with the *Hp* group, the antioxidant activity of GSH-Px and SOD in the *Hp*+Rapa and *Hp*+CE groups was increased (P<0.01) (Fig. 2**E, F**). These results show that CE can promote and regulate the occurrence of autophagy and the change of oxidative stress indicators in lung tissue cells.

**Fig. 3:**
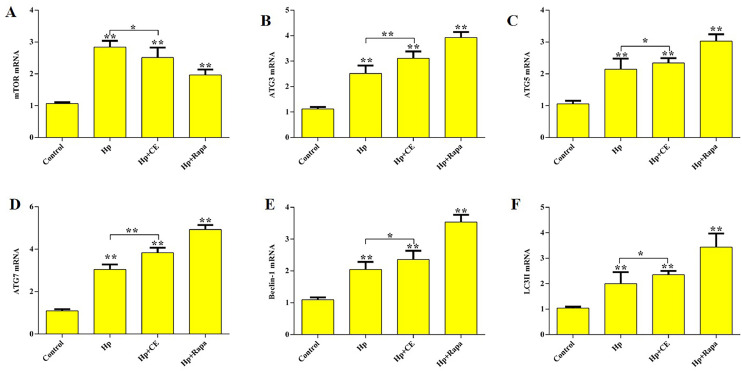
The effect of Hp on the transcriptional levels of autophagy-related genes in lung tissue cells. (*P<0.05, **P<0.01). (A) mTOR mRNA. (B) ATG3 mRNA. (C) ATG5 mRNA. (D) ATG7 mRNA. (E) Beclin-1 mRNA. (F) LC3II mRNA.

Further analysis via RT-PCR revealed significant changes in autophagy-related gene transcription levels. Compared to the control group, the *Hp* group showed a significant increase in the mRNA expression levels of mTOR, ATG3, ATG5, ATG7, Beclin-1, and LC3II (P<0.01). When compared to the *Hp* group, the *Hp*+CE and *Hp*+Rapa groups demonstrated a further significant increase in the mRNA expression levels of ATG3, ATG5, ATG7, Beclin-1, and LC3II (P<0.01), while the transcription level of mTOR was significantly decreased (P<0.01) (Fig. 3**A, B, C, D, E, F**). These results suggest that CE may modulate the transcription levels of key autophagy-related genes, thereby influencing *Hp*-induced lung injury.

### CE effects on the NLRP3/Caspase-1/IL-1β inflammatory pathway

**Fig. 4:**
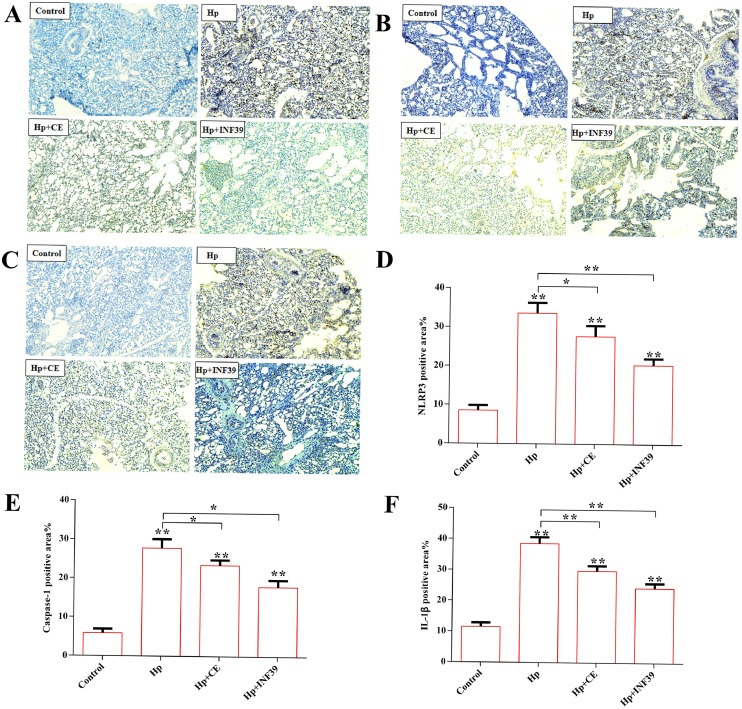
The effect of CE on the NLRP3/Caspase-1/IL-1β signaling pathway in Hp-induced lung injury. (A) Determination of NLRP3 protein in chicken lung tissue by immunohistochemical method (200x). (B) Determination of Caspase-1 protein in chicken lung tissue by immunohistochemistry (200x). (C) Determination of IL-1β protein in chicken lung tissue by immunohistochemistry (200x). (D) NLRP3 protein. (E) Caspase-1 protein. (F)IL-1β protein. (*P<0.05, **P<0.01).

We assessed the protein levels of NLRP3, Caspase-1, and IL-1β in chicken lung tissue via immunohistochemistry. Compared with the control group, the *Hp* group showed significantly higher protein expression of NLRP3, Caspase-1, and IL-1β (P<0.01). In comparison with the *Hp* group, the *Hp*+INF39 and *Hp*+CE groups exhibited markedly lower protein levels of NLRP3, Caspase-1, and IL-1β (P<0.01) (Fig. 4**A, B, C, D, E, F**). This indicates that CE may reduce *Hp*-induced pulmonary damage by inhibiting the protein expression of NLRP3, Caspase-1, and IL-1β, similar to the NLRP3 inhibitor INF39.

**Fig. 5:**
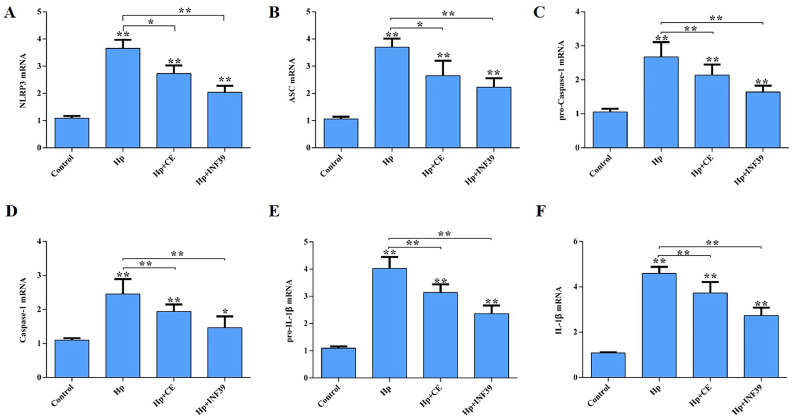
The effect of CE on genes related to the NLRP3/Caspase-1/IL-1β signaling pathway in lung injury of Hp-infected chicks. (A) NLRP3 mRNA. (B) ASC mRNA. (C) pro-Caspase-1 mRNA. (D) Caspase-1 mRNA. (E) pro-IL-1β mRNA. (F) IL-1β mRNA. (*P<0.05, **P<0.01).

RT-PCR was used to evaluate the expression of key genes in the NLRP3/Caspase-1/IL-1β signaling pathway in chicken lungs (Fig. 5**A, B, C, D, E, F**). Compared to the control group, the *Hp* group showed markedly elevated mRNA levels of NLRP3, ASC, pro-Caspase-1, Caspase-1, pro-IL-1β, and IL-1β (P<0.01). In contrast, these six genes were significantly downregulated in the *Hp*+CE and *Hp*+INF39 groups compared to the *Hp* group (P<0.01). These results suggest that in *Hp*-induced acute pulmonary injury in chicks, CE can modulate the expression of genes in the NLRP3/Caspase-1/IL-1β signaling pathway in the lungs.

## Discussion

The lung injury caused by *Hp* is a complex process that involves multiple cells and mechanisms. In the early stages of infection, the pathogen activates the host's innate immune system, which triggers a series of inflammatory responses (Crispo et al., 2019). Research shows that after infection with *Hp*, the expression of pro-inflammatory cytokines such as TNF-α and IL-1β is significantly increased in lung tissue. These cytokines recruit large numbers of immune cells, including neutrophils and macrophages, to the infection site. The reactive oxygen species (ROS) release and reactive nitrogen species (RNS) from these immune cells can further worsen tissue damage (Bustos et al., 2022; Wu et al., 2010; Albano et al., 2022).

In recent years, CE as a traditional Chinese medicine, has garnered attention for its unique chemical composition and diverse pharmacological effects, including antioxidant, anti-inflammatory, antibacterial and immunomodulatory (Choi et al., 2008). Its potential in preventing and treating lung injuries is also emerging as a focal point of interest. *Hp* as a significant pathogen responsible for avian respiratory diseases, poses a serious threat to the health and productivity of chickens by inducing a variety of diseases. Nevertheless, the protective effects of CE against bacterial-induced lung injuries have yet to be comprehensively explored. This study delves into the potential therapeutic effects of CE on lung injuries at the gene and protein levels. Initially, histological section observation revealed acute pulmonary damage, characterized by extensive inflammatory cell infiltration, thickened alveolar walls, narrowed alveolar spaces, and capillaries filled with red blood cells. ELISA assays indicated elevated expression levels of pro-inflammatory factors such as IL-1β, IL-18, and TNF-α, suggesting that *Hp* infection exacerbates inflammatory pathological damage in chicken lung tissue. Oxidative stress represents another pivotal mechanism underlying lung injury. Under normal physiological conditions, the body's oxidation and antioxidant systems exist in a state of dynamic equilibrium. However, during infections and inflammatory states, oxidative stress levels increase markedly (Bezerra et al., 2023). Excessive reactive oxygen and nitrogen species can compromise the integrity of cell membrane lipid bilayers, induce lipid peroxidation, generate harmful byproducts, and diminish the activity of antioxidant enzymes such as SOD and GSH-Px, thereby aggravating cellular damage (Juan et al., 2021). The alterations in SOD and GSH-Px levels noted in the present study are in agreement with the reported by Juan et al (Juan et al., 2021).

In bacterial pneumonia, increased LC3-II expression may indicate activated protective autophagy, though excessive autophagy can cause cell death (Wei et al., 2023). LC3 protein exists in two forms: LC3-I, the unmodified cytoplasmic form, and LC3-II, which is formed when LC3-I undergoes lipidation and integrates into the autophagosome membrane, serving as a direct marker of autophagosome formation (Tanida et al., 2004). The LC3I-to-LC3II ratio is a key indicator of autophagy levels. When autophagy is activated, LC3I is enzymatically cleaved to form LC3II, so higher LC3II levels and lower LC3I levels signify enhanced autophagy (Hansen et al., 2011). In this study, the mRNA expression of ATG3, ATG5, ATG7, Beclin-1, and LC3II was significantly upregulated in the *Hp* infection group. Similarly, in the CE and rapamycin treatment groups, the mRNA expression of these genes and the LC3II-to-LC3I ratio were significantly increased, indicating that CE treatment enhances autophagy in chick lung tissue cells induced by *Hp*.

The NLRP3/Caspase-1/IL-1β signaling pathway is a key player in many inflammatory responses. When activated, NLRP3 triggers Caspase-1 activation, which in turn processes pro-IL-1β into its mature form IL-1β (Kelley et al., 2019). Following *Hp* infection, genes and proteins in this pathway are upregulated in chicken lung tissue, and pro-inflammatory factors like TNF-α, IL-18, and IL-1β are secreted alongside autophagy induction. Research indicates that pharmacologically inhibiting the NLRP3 pathway with INF39 reduces the expression of NLRP3, Caspase-1, and IL-1β proteins (Shi et al., 2021). Our findings align with this, showing that such inhibition decreases the expression of these genes and proteins, thereby reducing the release of inflammatory factors. Similarly, in the CE treatment group, the expression trends of these genes and proteins mirrored those in the INF39 group. This suggests that CE may attenuate *Hp*-induced inflammatory damage in chicks by inhibiting the expression of these genes and proteins via the NLRP3/Caspase-1/IL-1β pathway.

In summary, CE has been shown to improve the pathological damage of pneumonia and reduce the secretion of pro-inflammatory factors. Our findings suggest that CE promotes autophagic activity, which in turn suppresses the activation of the NLRP3/Caspase-1/IL-1β signaling pathway, ultimately alleviating Hp-induced pulmonary damage in chickens.

## Institutional review board statement

In this research, all procedures were approved by the Experimental Animal Ethics Committee of Kunming University. Experiments were conducted in accordance with the Guidelines for the Ethical Review of Animal Welfare (EAE-GZU-2022-E021) and adhered to protocols approved by the Institutional Animal Care and Use Committee (IACUC).

## Funding information

This work was supported by Research Project on Risk Monitoring and Control Technology of Important Food-Borne Zoonotic Bacterial Diseases in Yunnan Province, 202407AB110017; Talent Introduction Project of Kunming University of China, XJ20230031; Yunnan Provincial Department of Education Project, ZX20240073.

## CRediT authorship contribution statement

**Bo Zhang:** Writing – original draft, Visualization, Methodology, Investigation, Formal analysis. **Bo Liao:** Writing – original draft, Visualization, Methodology, Investigation, Formal analysis. **Rou Sang:** Investigation, Formal analysis. **Rong Liao:** Investigation, Formal analysis. **Dengyao Xu:** Investigation, Formal analysis. **Yanhong Lv:** Investigation, Formal analysis. **Fangjie Li:** Visualization, Investigation, Formal analysis. **Ke Li:** Writing – review & editing, Validation, Software, Resources, Project administration, Methodology, Investigation, Funding acquisition, Conceptualization. **Aiguo Xin:** Writing – review & editing, Visualization, Software, Resources, Project administration, Methodology, Investigation, Funding acquisition, Formal analysis, Conceptualization.

## Disclosures

The authors declare the following financial interests/personal relationships which may be considered as potential competing interests: Bo Zhang reports financial support was provided by Yunnan Academy of Animal Husbandry and Veterinary Sciences. If there are other authors, they declare that they have no known competing financial interests or personal relationships that could have appeared to influence the work reported in this paper.
